# Investigating Heritage Language Processing: Meaning Composition in Chinese Classifier-Noun Phrasal Contexts

**DOI:** 10.3389/fpsyg.2021.782016

**Published:** 2021-12-17

**Authors:** Fei Li, Xiangfei Hong, Zhaoying He, Sixuan Wu, Chenyi Zhang

**Affiliations:** ^1^School of Foreign Languages, Shanghai Jiao Tong University, Shanghai, China; ^2^Shanghai Mental Health Center, Shanghai Jiao Tong University School of Medicine, Shanghai, China

**Keywords:** lexical-semantic processing, chinese as a heritage language, ERP, N400, LPC

## Abstract

The aim of the present study was to investigate how Chinese-Malay bilingual speakers with Chinese as heritage language process semantic congruency effects in Chinese and how their brain activities compare to those of monolingual Chinese speakers using electroencephalography (EEG) recordings. To this end, semantic congruencies were manipulated in Chinese classifier-noun phrases, resulting in four conditions: (i) a strongly constraining/high-cloze, plausible (SP) condition, (ii) a weakly constraining/low-cloze, plausible (WP) condition, (iii) a strongly constraining/implausible (SI) condition, and (iv) a weakly constraining/implausible (WI) condition. The analysis of EEG data focused on two event-related potential components, i.e., the N400, which is known for its sensitivity to semantic fit of a target word to its context, and a post-N400 late positive complex (LPC), which is linked to semantic integration after prediction violations and retrospective, evaluative processes. We found similar N400/LPC effects in response to the manipulations of semantic congruency in the mono- and bilingual groups, with a gradient N400 pattern (WI/SI > WP > SP), a larger frontal LPC in response to WP compared to SP, SI, and WI, as well as larger centro-parietal LPCs in response to WP compared to SI and WI, and a larger centro-parietal LPC for SP compared to SI. These results suggest that, in terms of event-related potential (ERP) data, Chinese-Malay early bilingual speakers predict and integrate upcoming semantic information in Chinese classifier-noun phrase to the same extent as monolingual Chinese speakers. However, the global field power (GFP) data showed significant differences between SP and WP in the N400 and LPC time windows in bilinguals, whereas no such effects were observed in monolinguals. This finding was interpreted as showing that bilinguals differ from their monolingual peers in terms of global field power intensity of the brain by processing plausible classifier-noun pairs with different congruency effects.

## Highlights

- The manipulation of semantic congruency elicited similar N400/LPC patterns between bilinguals and monolinguals.
- ERP data indicates Chinese-Malay early bilingual speakers predict and integrate semantic information to the same extent as monolingual Chinese speakers.
- GFP data showed significant differences between SP and WP in the N400/LPC time windows in bilinguals, whereas no such effects were observed in monolinguals.

## Introduction

Over several decades, the fact that two or more languages can coexist in one mind has sparked the interest of many researchers. However, there is still no agreed-upon answer to the central question: How distinguishable are the neural representations of the first language (L1) in native speakers from those in bilingual speakers, i.e., people who masters, understands, and speaks more than one dialect or language (Fabbro, 1999). Studies using brain imaging techniques (see Cargnelutti et al., 2019; Połczyńska and Bookheimer, 2020, 2021, for reviews) have demonstrated that a number of key variables affect the degree of neural representation overlap between languages, including the age of acquisition, proficiency level, the amount of language exposure, the way of language learning (implicit versus explicit), and the linguistic distance between languages (typological similarity). More similar neural representations should be observed, when languages share more identical acquisition and linguistic variables, that is, they were acquired early in life (i.e., before the critical period, see DeKeyser, 2013, for recent discussions), learned informally, and when they are spoken with a high degree of proficiency (Newman et al., 2012). The primary goal of the present study was to examine how similar the neural response pattern elicited by Chinese classifier-noun phrases in highly proficient bilingual speakers compared with that in Chinese monolingual speakers.

The bilinguals in this study were Chinese-Malay bilinguals in Malaysia who were heritage speakers of Chinese. Heritage speakers as a homogeneous population is difficult to define because each heritage speaker has his or her complex language experience that is affected by language exposure, speaker diversity, literacy, community support, motivation and so on, all of which interact to influence the development and end state of the individual’s heritage language. However, there are some commonalities among heritage speakers. They are often the children of immigrants whose parents have moved from a country of origin speaking one language to a different country speaking another; the children of immigrants acquire their heritage language (L1) naturalistically in the home environment, and also acquire a second language (L2) either simultaneously, or at a relatively later age, usually before the onset of adolescence (Van Deusen-Scholl, 2003; Montrul, 2016). Usually, the L2 becomes the dominant majority language and the L1 weakens. In this sense, almost all heritage speakers are facing the problems of “incomplete L1 acquisition” and “L1 attrition” (Polinsky, 2006; Montrul, 2008, 2016; Schmid and Köpke, 2009; Benmamoun et al., 2013; Scontras et al., 2015; Montrul and Silva-Corvalán, 2019; Gallo et al., 2021). Incomplete L1 acquisition means that the heritage speakers did not have an opportunity to reach age-appropriate mastery of the L1. However, it is not caused by a deficient ability to fully acquire the L1, but instead it is due to the fact that some specific properties of the heritage language remain absent from the heritage speakers’ language environment, either because their parents do not use them or because the heritage speakers do not have opportunities to use them (Montrul, 2008; Montrul and Silva-Corvalán, 2019). L1 attrition means deterioration or even loss of a linguistic property after fully attained, due to intensive L2 exposure and reduced L1 use (Montrul, 2008; Schmid and Köpke, 2009; Gallo et al., 2021). Young heritage speakers’ L1 knowledge is likely to reflect incomplete acquisition and language attrition “simultaneously or sequentially” (Montrul, 2008, p. 21). This process of language acquisition often results in unbalanced bilingualism, with the greatest proficiency being in the L2, at the expense of the heritage L1(Montrul, 2016).

The imbalance in the bilingual language system has sparked much of the research interest in a deeper understanding of the human language processing. Many past studies have revealed widespread differences in the phonetics/phonology, morphology, syntax, and lexical-semantic areas in which bilingual speakers have deficiencies when compared to monolingual speakers, particularly those who are captured in offline and productive language tasks (e.g., Montrul, 2006, 2016; Benmamoun et al., 2013; Flores and Barbosa, 2014; Casillas and Simonet, 2016). However, studies using online measures, such as event-related potentials (ERPs, Weber-Fox and Neville, 1996; Braunstein et al., 2012; Kasparian and Steinhauer, 2016), have uncovered greater similarity between monolingual and bilingual lexico-semantic processing than previously assumed, especially when bilinguals have a high level of target language proficiency. These similarities raise the question of whether bilingual language acquisition is truly “deficit,” or whether the observed differences in offline measures are a manifestation of some other non-linguistic factors such as tolerance for expression diversity, self-confidence in their bilingual abilities, or decision-making, at least in highly proficient heritage speakers.

### Chinese Heritage Speakers in Malaysia

Chinese-Malay heritage speakers is one population of bilinguals that very few researchers in psycholinguistics have addressed. They are generally second- or third-generation immigrants who grow up in Chinese Malaysian households. Chinese Malaysians (often described as “Malaysian Chinese”) accounts for around 28% of 30.9 million national population and is the second-largest linguistic group in Malaysia (Tan C.-B, 1997; Vollmann and Soon, 2018). The majority of the Chinese immigrants to Malaysia were from south provinces in China such as Fujian, Guangdong, and Jiangxi. Thus, Chinese Malaysians generally have multiple identities: the identity of a specific Chinese dialect group (e.g., Foochow, Hokkien, and Cantonese), the unique racial and cultural identity of the overseas Chinese, and the national identity of Malaysian (Tan M. G, 1997). However, they use Mandarin (locally called huá yǔ) as the common Chinese language, and in Chinese education in Malaysia, the medium of instruction is Mandarin rather than a speech-group dialect (Tan C.-B, 1997; Wang, 2009). In daily life, Chinese Malaysians communicate in various Chinese dialects, Mandarin, Malaysian English and, if necessary, Malay or Bahasa Pasar (a common language composed mainly of Malay with some Chinese dialects, English and Tamil components). In Malaysia, there are different Chinese speech-groups and regional Chinese identities. The main contrast is between the least localized Chinese and the Baba (a Malay-speaking group of Chinese in Malaysia), although, in fact, the localization of different subgroups of Chinese Malaysians differs only in a matter of degree (Tan C.-B, 1997; Vollmann and Soon, 2018). As pointed out by Phooi-Yan Lee and Ting (2016), language and education influence the perception of Chinese identities in Malaysia. Heritage speakers who went to a Chinese-medium school often shows a stronger Chinese identity, the tendency to use Chinese, and better Chinese proficiency and literacy. In our research, we have a group of heritage speakers with more Chinese identity: They went to a Chinese primary school in Malaysia where education takes place in Chinese and English, with Malay as L2; after that, they attended either a public high school (Chinese-medium) finishing with the national high school certificate, or a private Chinese high school (Chinese- or English-medium, for more details about the schooling system in Malaysia, see Wang, 2016). During the experiment, all of them have attended a university in mainland China for at least 1 year. In these situations, the experiences of the heritage speakers in our study may bring them closer to “complete,” native level to Chinese, compared to other “incomplete,” unbalanced heritage bilinguals such as Spanish heritage speakers in assimilationist communities in America (Montrul, 2006, 2008).

### The N400 and the Late Positive Complex

Most ERP studies of language have focused on the N400, a centroparietal negativity peaking around 400 ms after the stimulus onset, which has proven to be a reliable and consistent measure for the processing of meaning (Kutas and Hillyard, 1980; Kutas and Federmeier, 2011; Federmeier, 2021). The amplitude of the N400 varies as a function of contextual constraint and cloze probability (refers to the probability of a sample of participants to use a specific word to complete a sentence from which a necessary sentence completion is omitted, such as “I drink my coffee with sugar and —”), showing a reduced amplitude when the target word and contextual information are consistent, and an increased amplitude when the linguistic features of the target stimulus do not fit the context (Kutas and Hillyard, 1980, 1984; Van Berkum et al., 1999, 2003; Federmeier et al., 2007). The classic N400 effect is considered to reflect contextually facilitated lexical access to long-term semantic memory, as well as reduced difficulty in integrating new information with prior context (Hagoort, 2003; Lau et al., 2008; Brouwer et al., 2012; Molinaro et al., 2012; DeLong et al., 2014; Brothers et al., 2015; Li et al., 2021).

With regard to ERPs following the N400 time window, many studies report a Late Positive Complex (LPC), a positive deflection beginning around 500 ms after stimulus onset, typically with a frontocentral maximum after unexpected but plausible target words in a constraining sentence context (Federmeier et al., 2007; Urbach and Kutas, 2010; Delong et al., 2011; Thornhill and Van Petten, 2012), or with a centroparietal maximum after a deeply implausible stimulus (Kolk et al., 2003; Kim and Osterhout, 2005; Van Herten et al., 2006; Kuperberg, 2007; Bornkessel-Schlesewsky and Schlesewsky, 2008; Van De Meerendonk et al., 2010).

In few research with phrase structures, researchers found late LPC effects, with a frontal distribution by “unnatural” but plausible combinations (e.g., a lovely monster, Molinaro et al., 2012; Li et al., 2021), or with a posterior distribution by anomalous combinations (e.g., the wooden *dove*, Schumacher, 2013). Others studies focusing on syntactic have also found a frontocentral P600 effect, as an index of ambiguity resolution and/or syntactic complexity, or a centroparietal P600 effect, as an index of syntactic violations or syntactic processing difficulties (Friederici et al., 2002; Kaan and Swaab, 2003).

The functional nature of the LPC is still unclear. It has been hypothesized that the more frontally distributed LPC reflects the successful updating of the comprehender’s current mental model with new unexpected but plausible/possible input, which entailed the inhibition of incorrectly selected lexical items (Kutas, 1993; Federmeier et al., 2007; Thornhill and Van Petten, 2012; Van Petten and Luka, 2012; Wlotko and Federmeier, 2012; DeLong et al., 2014; Kuperberg and Wlotko, 2018; Ness and Meltzer-Asscher, 2018). The more posteriorly distributed LPC, by contrast, reflects the failure to update new unexpected and deeply implausible/impossible input into the comprehender’s existing mental model, which is frequently interpreted as signaling reanalysis of previous mental representation in attempts to revise or repair the current model (Kim and Osterhout, 2005; Van De Meerendonk et al., 2010; Kuperberg and Wlotko, 2018; Leckey and Federmeier, 2020). In the framework of the investigation about the “semantic illusion” phenomenon, the interpretation of the LPC can be further refined with respect to functionally dissociable processes linked to the biphasic N400/LPC effect: an earlier immediate congruency effect responsive to the semantic match between the target word and its preceding context, reflected by the N400, and later more controlled and attention-driven processes which may contribute to updating, revising/reanalyzing and reorganizing information in a mental representation, reflected by the LPC (Brouwer et al., 2012; Kuperberg and Wlotko, 2018; Rabovsky et al., 2018).

### The N400 and the Late Positive Complex in Bilingual Research

As compared to monolinguals, prior research has found reduced amplitude and/or delayed peak latency of the N400 to semantic anomalies in late bilinguals (Ardal et al., 1990; Weber-Fox and Neville, 1996; Hahne, 2001; Friederici et al., 2002; Hahne and Friederici, 2002) but not in early bilinguals (Weber-Fox and Neville, 1996) or proficient late bilinguals (Braunstein et al., 2012). Furthermore, some studies have found a different N400 pattern between mono- and bilingual groups not only to semantic anomalies but also to semantically correct target words, by showing correct stimuli elicited a larger negativity in the bilingual group than the monolingual group (Hahne, 2001; Hahne and Friederici, 2001; Newman et al., 2012). These effects were modulated by the age of L2 exposure and L2 proficiency (Weber-Fox and Neville, 1996; Kotz, 2001; Kotz and Elston-Güttler, 2004; Mueller, 2005; Liang and Chen, 2020), even in a separate way (Newman et al., 2012; Kasparian and Steinhauer, 2016). For instance, Newman et al. (2012) investigated the influence of L2 proficiency on N400 effects elicited by lexical semantic anomalies in English sentences. In this study, the researchers found that N400 amplitudes to semantically plausible target words were larger for subjects with lower English proficiency, in both monolingual English speakers and late learners with Spanish as L1, suggesting an independent influence of language proficiency on N400 amplitudes. Researchers pointed differences between mono- and bilingual processing to slowed, less automatized access to lexical information and a reduced speed of semantic analysis/integration in bilinguals (Ardal et al., 1990; Weber-Fox and Neville, 1996; Moreno and Kutas, 2005; Mueller, 2005), or/and to less certain vocabulary knowledge and use in the target language due to a weaker word-conceptual link (Kotz, 2001;Hahne and Friederici, 2002; Kotz and Elston-Güttler, 2004; van Heuven and Dijkstra, 2010).

Although only few studies have investigated the LPC in a bilingual population, controversial findings have been reported (Martin et al., 2013; Foucart et al., 2014; Kasparian and Steinhauer, 2016; Zheng and Lemhöfer, 2019). Kasparian and Steinhauer (2016) found an enhanced late posterior positivity (labeled as P600 in Kasparian and Steinhauer’s article; see Van Petten and Luka, 2012; Kuperberg and Wlotko, 2018; Leckey and Federmeier, 2020, for discussions about the relationship between LPC and P600) to lexical-semantic violations in L1 Italian attriters (note that all heritage speakers are, in a broad sense, L1 attriters, see Gallo et al., 2021, for detailed discussions), compared to adult Italian L2 learners and to Italian monolingual speakers, regardless of language proficiency. The researchers attributed that effect to increased conflict-monitoring and second thought processes specifically in attriters. In line with the results of Kasparian and Steinhauer (2016), Zheng and Lemhöfer (2019) argued that early L2 learners show the same posterior LPC effects as the native speakers do, but only when L2 learners find the conflict in syntactically correct but semantically implausible sentences. If they could not detect the conflict, the LPC effect in response to semantic implausibility would be largely attenuated in L2 learners. With regard to the frontal LPC, some studies suggest that bilingual speakers do not anticipate to the same extent as monolingual speakers, reflected by a reduced LPC (Martin et al., 2013), whereas others found that bilinguals are able to anticipate incoming words in a similar manner as their monolingual peers (Foucart et al., 2014). Kaan (2014) reviewed previous ERP studies on this topic and proposed that mono- and bilingual speakers do not differ in the nature of the predictive mechanisms, but in factors that drive these mechanisms. Differences between the groups could be attributed to a variety of changeable factors, such as the frequency information stored, accuracy and consistency of lexical representations, and interlingual competition; those who are more exposed to the target language and have greater proficiency in that language are likely to have more firmly anchored target language information in memory, more easily lexical access, and more enhanced ability in monitoring different languages at the same time. Indeed, some very current ERP studies have shown that predictive abilities in bilingual speakers are not unchanged, but increase with increasing language experience and language use, especially, when the control ability of bilinguals is strong (Zirnstein et al., 2018), or when a bilingual’s languages are typologically similar (Foucart et al., 2014).

### Chinese Classifier-Noun Phrase as the Representative of Language Processing

The current study examined whether language processing engenders similar neural responses in Chinese monolingual speakers and fluent Chinese-Malay bilingual speakers. We selected the Chinese classifier-noun phrase as the representative of language processing for two reasons. Firstly, a classifier-noun phrase can be used to investigate semantic congruency effects in a minimal phrase structure context. In Chinese, classifiers denote some salient perceived or imputed characteristics of the entity to which their pairing nouns refer, such as humanness, animacy, shape/form, size, function or idiosyncratic (Erbaugh, 1986; Lakoff, 1986; Aikhenvald, 2006; Bi, 2017; Kemmerer, 2017). They coerce the interpretation of the noun they classify by eliminating other possible interpretations and combine with the noun to create a meaning toward an individual, a kind, or an event reading (Pustejovsky, 1991; Huang and Ahrens, 2003). For example, the classifier běn can classify bound print matter such as book, and refers to a book as individuum. This type of semantic coercion can be used to investigate congruency effects between classifiers and nouns. In particular, there are semantically relatively defined and restricted classifiers, such as the classifier zhǎn associated with lamps. At the same time, there are classifiers (e.g., kē) which can denote a range of objects (e.g., kē can denote small objects like beans, hearts, pearls, teeth, diamonds, etc., as well as objects appearing to be small, such as stars and planets). This allowed us to investigate whether upon seeing a classifier, comprehenders would use it as a linguistic marker and thus predict only nouns belonging to its membership, similar to an adjective as a predictable marker of its possible following nouns in adjective noun phrases (e.g., Molinaro et al., 2012).

Secondly and more importantly, classifiers are a good tool for investigating how structural features of languages affect attribute accessibility and object categorization. Chinese classifiers have different degrees of typicality of individual nouns (Zhang and Schmitt, 1998; Gao and Malt, 2009; Speed et al., 2021, but see Saalbach and Imai, 2007, for a different view). Typicality is an important property of a category, relating to graded goodness of example in a categorical hierarchy (Rips et al., 1973; Rosch and Mervis, 1975). Not every member of a category is regarded as a good example; on the contrary, members lie on a spectrum of categorical goodness. While some items were judged as typical examples or prototypes, other items were judged as atypical members. For instance, pearls are more often judged as typical members of the category restricted by the classifier kē than are salt grains, since kē is most typically paired with small but not extremely small objects. The typicality gradient is generally considered to reflect the internal membership structure of a concept, reflecting featural correlations between different items (McRae et al., 1999) or strong links between nodes in a hierarchical manner (Collins and Loftus, 1975). Using classifier noun phrases provides a meaningful insight into how people link features in semantic networks, and how they categorize the world through their language.

### The Present Study

In the current study, we aimed to determine whether the findings of our previous ERP study (Li et al., 2021) on brain activity patterns in Chinese monolingual speakers in relation to the processing of Chinese classifier-noun phrases were present in Chinese-Malay bilingual speakers, as indexed by the N400 and the post-N400 LPC.

**FIGURE 1:**
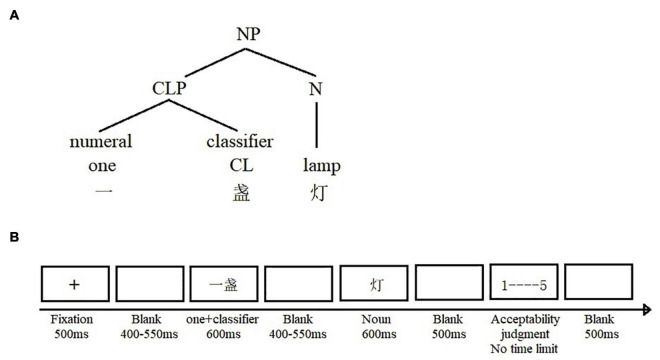
**(A)** The structure of the classifier-noun phrase in the form of “numeral + classifier + noun.” NP, noun phrase; CLP, classifier phrase; CL, classifier; N, noun. The classifier and the noun form a local phrase structure, and **(B)** example trial and timing of the experiment.

As in our previous study (Li et al., 2021), semantic congruencies between classifiers and nouns in Chinese classifier-noun pairs were manipulated, resulting in four conditions (see Figure 1A): (i) a strongly constraining/high-cloze, plausible (SP) condition, (ii) a weakly constraining/low-cloze, plausible (WP) condition, (iii) a strong constraining/implausible (SI) condition, and (iv) a weakly constraining/implausible (WI) condition. The predictions of the present study are straightforward. If bilinguals, having acquired Chinese since birth and having lived in an exclusively Chinese environment until adulthood, remain native-like in their Chinese lexical-semantic processing despite their intensively L2 use, we would expect to observe a native-like N400/LPC pattern in the bilingual group. More specifically, we expected a graded modulation of the N400 component for the four conditions, with SP elicits the smallest N400, SI and WI the largest N400, whereas WP elicits an N400 of intermediate amplitude. In contrast, bilinguals may not show native N400 responses, especially for the semantically fine-grained WP condition; this could point to weakening word-conceptual link that makes lexical access and semantic integration less efficient in the bilingual brain, reflecting a larger N400 in bilinguals compared to monolinguals in response to the WP condition (Kotz, 2001;Hahne and Friederici, 2002; Kotz and Elston-Güttler, 2004; van Heuven and Dijkstra, 2010). In addition, previous evidence shows that target words with the same low cloze probability do not differ in N400 activity, regardless of whether word meaning had already been pre-activated in a relatively high-constraint sentence context or not (Federmeier et al., 2007; Quante et al., 2018), at least when animacy violations are not present (Szewczyk and Schriefers, 2011; Kuperberg and Wlotko, 2018). This finding is considered as evidence that the N400 does not reflect the processing cost of prediction violations (see review by Van Petten and Luka, 2012). Consistent with prior work with sentence contexts (Federmeier et al., 2007; Quante et al., 2018) and unlike other studies with animacy violations (Szewczyk and Schriefers, 2011; Kuperberg and Wlotko, 2018), our previous study with monolinguals (Li et al., 2021) did not find any context effect for implausible nouns in classifier-noun phrases where contexts were either categorized as high constraint (the SI condition) or as low constraint (the WI condition). In the present study, we examined whether there are distinguished context effects associated with features provided by preceding classifiers between mono- and bilingual speakers. Finally, we also investigated whether the LPC component could be observed by semantic conflicts in bilinguals. Specifically, we had a specific hypothesis that predicted a more frontally distributed LPC for the unexpected but plausible WP condition, as shown in our previous study with Chinese monolinguals (Li et al., 2021), if bilinguals processed conflicts in classifier-noun phrases similarly to their monolingual peers.

## Materials and Methods

### Participants

Twenty-six Chinese-Malay bilinguals (7 females) with a mean average age of 21.2 years (*SD*: 1.72, range: 17–24 years) participated in this experiment. Their results were compared with the results of 32 monolingual speakers of Chinese (23 females) with a mean age of 22 years (*SD*: 2.68, range: 18–30 years) which have been reported in our previous ERP study (Li et al., 2021). No significant difference was present in age between bilinguals and monolingual (*p* > 0.05). All bilinguals reported that they used Chinese more than half of the time daily. Their mean length of stay in China was 28.8 months (*SD* = 16.7). Fifteen of the 26 bilingual participants had taken the second highest Chinese language proficiency test, HSK (hàn yǔ shuǐ píng kǎo shì) Level 5, before enrolling in the University. The HSK, designed by the Ministry of Education Agency hàn bàn (the Chinese name of the Chinese Language Council), is an international standardized test of Chinese language proficiency. It assesses non-native Chinese speakers’ abilities in using the Chinese language in their daily, academic and professional lives (Teng, 2017). Among the 15 heritage participants in our study who took the HSK, the mean HSK score was 267.5 (range: 211–288) of a total score of 300, indicating participants’ high level of Chinese proficiency. According to Zhang et al. (2020), the HSK yields the most consistent and reliable results with the largest effect size to examine a student’s Chinese proficiency level, compared to a self-report measure of years of instruction in Chinese, a reading comprehension test, and a Chinese character recognition test in their study. All participants were undergraduate or graduate students at a University in Shanghai, right-handed according to self-report, with no reading disabilities and with normal or correct-to-normal vision. Participants were paid 100 RMB for their participation. Signed informed consent was obtained from each participant before the experiment. The experimental protocol complied with the research ethics requirements of the Declaration of Helsinki and with the Research Ethics Committee within the School of Foreign Languages, Shanghai Jiao Tong University.

### Material Construction

The materials and design employed were identical to that in Li et al. (2021) to allow for the closest comparison between the mono- and bilingual groups. The critical materials contained 72 classifiers which are commonly used noun classifiers in Mandarin Chinese and were selected from Chinese Proficiency Test Syllabus Levels 4–5 (Confucius Institute Headquarters, 2009) and Modern Chinese eight hundred words (Lü, 1999). Half of these classifiers were strongly constraining classifiers and the other half were weakly constraining. Every classifier appeared twice throughout the whole experiment, once it would be paired with a plausible noun and once with an implausible noun. The number of the characters per noun varied between one and two and was counterbalanced across conditions. The classifiers across the constraint conditions were matched for word frequency according to Dictionary of Modern Chinese Frequency (Language and Teaching Institute of Beijing Linguistic College, 1986), and the number of strokes. Details about the materials and norming procedures used to create the stimuli are described in Li et al. (2021), or can be found in the Supplementary Materials. The examples, characteristics of the four conditions are listed in Table 1.

**TABLE 1:** Example of the stimuli for each condition and their characteristics (means are shown with SD in parentheses).

Conditions		Classifier-noun pairs	Classifier			Noun		
			Constraint rating	Frequency	Num. of strokes	Cloze probability	Frequency	Num. of strokes
**Strong**	**pl.-HC**	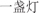	2.7 (0.33)	0.01 (0.01)	8.44 (2.62)	0.45 (0.26)	0.02 (0.03)	11.11 (4.60)
	**impl.**	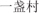				0.00 (0.00)	0.02 (0.02)	12.31 (4.91)
**Weak**	**pl.-LC**	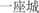	3.9 (0.32)	0.01 (0.01)	7.72 (2.95)	0.04 (0.03)	0.02 (0.02)	11.31 (5.26)
	**impl.**	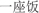				0.00 (0.00)	0.01 (0.01)	11.50 (4.51)

*Strong, strongly constraining; weak, weakly constraining; pl.-HC, plausible, high-cloze noun; pl.-LC, plausible, low-cloze noun; impl., implausible noun; Num., number. Examples: 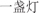 – yī zhǎn dēng, one classifier lamp – ‘a lamp’; 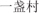 – yī zhǎn cūn, one classifier village; 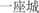 – yī zuò chéng, one classifier city – ‘a city’; 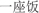 – yī zuò fàn, one classifier rice.*

### Procedure

Participants were seated approximately 100 cm from the computer screen in a sound-attenuating booth. E-prime 2.0 software (Psychology Software Tools, Pittsburgh, PA, United States) was used to implement the experimental paradigm. The EEG was continuously recorded when participants performed the experimental task. Each trial began with the presentation of a central fixation cross for 500 ms, followed by a blank screen presented randomly between 400 and 550 ms. First, “the numeral (one) + classifier” (e.g., yī zhǎn – one classifier) was presented centrally on the computer screen for 600 ms, followed by a random inter-stimulus interval between 400 and 550 ms. An irregular interstimulus interval was used in order to abolish alpha amplitude and phase consistency at target stimulus onset (cf. Hanslmayr et al., 2011). Then, the noun (e.g., dēng – lamp) was presented centrally for 600 ms, followed by an additional 500 ms blank screen before the start of next trial (see Figure 1B for details). A 5-point Likert-type scale from 1 (totally unacceptable) to 5 (perfectly acceptable) appeared after each phrase on the screen. Participants used a mouse to click on a score from the 1-5 acceptability scale to indicate their response. A rating scale rather than a binary acceptability judgment task was used, in order to assess whether conditions yield graded behavioral response patterns and whether these were related to ERP patterns. This scale remained on the screen until the decision was made. All trials were evenly divided into nine blocks, each consisting of 16 trials. Participants were given a break after each block. A practice session with 12 items was provided, and the whole experiment lasted for one and a half hours. To reduce artifacts caused by eye movements and eye blinks, participants were instructed to remain as still as possible with their eyes fixed at the center of the screen throughout each phrase trial, and to refrain from blinking as much as possible when stimuli were presented and were encouraged to rest during the inter-trial interval.

### Electroencephalography Recording and Preprocessing

During the experiment, EEG was recorded continuously from 64 Ag/AgCI electrodes by the NeuroScan, which were mounted on an elastic cap according to the 10–20 system (Jasper, 1958). In addition to the scalp sites, two electro-oculogram (EOG) channels were placed above and below the left eye (VEOG), and two at the outer canthi of both eyes (HEOG), as well as 2 electrodes placed on the left and right mastoids (M1 and M2). All electrodes were referenced online to a reference electrode placed between Cz and CPz. Impedances were kept below 8 kΩ. EEG signals were amplified and digitized at 1,000 Hz sampling rate and filtered online with a band-pass of 0.05–400 Hz. Data preprocessing was performed in Matlab-based (Version: R2014a) EEGLAB (Version: 13.5.4b) and ERPLAB (Version: 6.0) toolboxes.

Offline, raw continuous EEG data first went through a band-pass filter (0.1–40 Hz, a two-way Butterworth filter with zero phase shift; roll-off slope: 12 dB/oct), followed by a Parks-McClellan notch filter at 50 Hz. After that, EEG data were down-sampled to 250 Hz. To correct ocular artifacts, independent component analyses (Infomax algorithm) was performed and ocular components were identified by visual inspection. Typically, one or two components were removed for each participant. EEG data were then re-referenced to the algebraic average of the two mastoid electrodes. Continuous EEG data were then segmented into epochs from 200 ms pre-stimulus onset to 1,000 ms post-stimuli onset, with the 200 ms pre-stimulus period as baseline for baseline correction. Artifact detection was performed for all EEG epochs, according to the following criteria: (i) the maximally allowed amplitude difference for all EEG channels within a moving window (width: 200 ms; step: 50 ms) should not exceed 150 μV; (ii) the maximally allowed absolute amplitude for all EEG channels throughout the whole epoch should not exceed 100 μV. Artifact-contaminated target trials were rejected before averaging. On average, there were 33 and 35 trials (out of the total 36 trials) remaining per condition for monolinguals and bilinguals, respectively, after preprocessing.

### Data Analyses

Event-related potentials time-locked to the onset of nouns were computed for each participant and for each condition, with a baseline correction of −200 to 0 ms. Grand-averaged waveforms were derived by averaging individual ERPs. Statistical analyses were conducted on mean ERP amplitudes for the critical words, using a spatiotemporal region-of-interest (ROI) approach. Consistent with our previous study (Li et al., 2021), N400 amplitudes were measured based on the averaged waveforms across five centro-parietal electrode sites (Cz, CPz, Pz, CP1, and CP2), where such responses are characteristically most prominent (e.g., Kutas and Hillyard, 1984; Federmeier et al., 2007; Kutas and Federmeier, 2011; Brothers et al., 2015; Fleur et al., 2020). To evaluate possible LPC differences between anterior and centro-parietal electrode sites, we averaged voltages across the five centro-parietal electrodes as well as five anterior electrode sites (F3, Fz, F4, FC1, and FC2) in the 500–700 ms (post-N400) time window, according to previous research which identified frontal LPC components in response to unpredicted but plausible items in constrained contexts (Martin et al., 2013; Chou et al., 2014; DeLong et al., 2014; Brothers et al., 2015; Freunberger and Roehm, 2016; Quante et al., 2018; Fleur et al., 2020). For the N400, a two-way ANOVA including one within-subject factor of Condition (SP vs. WP vs. SI vs. WI) and one between-subject factor of Group (bilingual vs. monolingual) was conducted. For the LPC, a three-way ANOVA with one more within-subject factor of ROI (frontal vs. centro-parietal) was conducted. Follow up ANOVAs were conducted when significant interactions with Condition were presented. The Greenhouse–Geisser correction (Greenhouse and Geisser, 1959) was applied when appropriate, and in this case, the uncorrected degrees of freedom but corrected *p*-values were reported. Significant interactions in the follow up ANOVAs were decomposed by using *post hoc t*-tests with Bonferroni correction. We report *F*-values, degrees of freedom (df), *p*-values, and partial eta-squared ($\eta_{\text{p}}^{2}$) for an estimation of effect sizes. We used IBM SPSS STATISTICS version 22 for all statistical analyses.

In order to consider the overall differences in electric potential for all EEG electrodes, we also calculated the global field power (GFP) for each condition and each group. As a reference-independent measure, GFP represents the spatial standard deviation of the potential across the entire scalp at each sampling point of the epoch window (Lehmann and Skrandies, 1980). For each subject, GFP values of each condition were translated into *z*-scores with the pre-stimulus 200 ms as baseline. Significant differences (*p* < 0.05) in *z*-scores between conditions that persisted for at least 20 ms in either group were highlighted (Rao et al., 2010; Zhang et al., 2011). Raw data and Material are available in OSF^1^.

## Results

### Behavioral Results

With mean acceptability scores of 4.85 (*SD* = 0.62), 4.73 (*SD* = 0.83), 1.15 (*SD* = 0.53), and 1.20 (*SD* = 0.62) in monolinguals, and of 4.45 (*SD* = 1.22), 4.27 (*SD* = 1.35), 1.26 (*SD* = 0.69), and 1.31 (*SD* = 0.80) in bilinguals for the SP, WP, SI, and WI conditions, respectively, indicating that both groups of the participants reliably accepted the plausible classifier-noun pairs and rejected the implausible classifier-noun pairs. A repeated-measures ANOVA with within-subject factor Condition (SP vs. WP vs. SI vs. WI) and between-subject factor Group (bilingual vs. monolingual) revealed a significant main effect of Group [*F*(1,56) = 6.42, *p* = 0.014, $\eta_{\text{p}}^{2}$ = 0.103], a significant main effect of Condition [*F*(3,168) = 1406.34, *p* < 0.001, $\eta_{\text{p}}^{2}$ = 0.962], and a Condition × Group interaction [*F*(3,168) = 9.30 *p* = 0.003, $\eta_{\text{p}}^{2}$ = 0.142]. *Post hoc* comparisons with Bonferroni correction showed the SP and WP conditions were rated lower in bilinguals than monolinguals (SP: *p* = 0.0015; WP: *p* < 0.001), while no such group differences were observed in the SI and WI conditions (both *p* > 0.19). Thus, it could be conceivable that the bilinguals in our study were less certainty with the associative relations between classifiers and nouns in the plausible SP and WP conditions.

### Event-Related Potential Data

#### The N400

**FIGURE 2:**
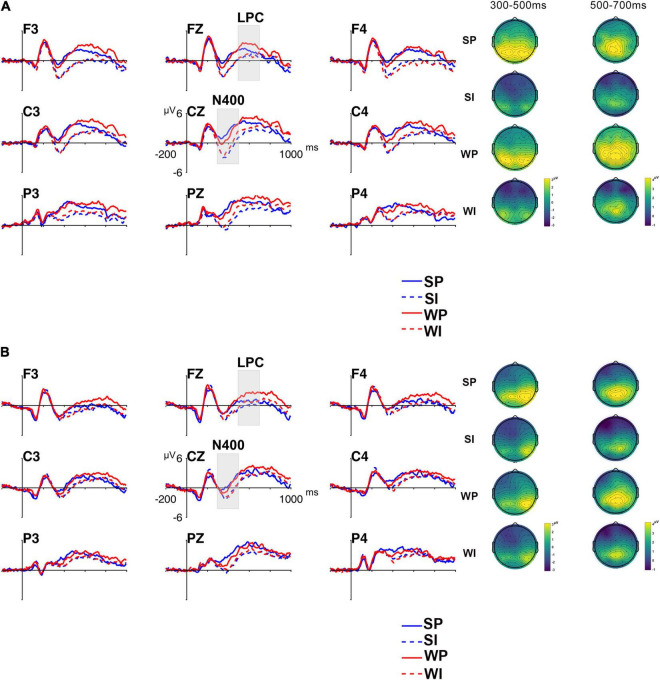
Grand-average ERP waveforms for critical conditions over representative electrodes and topographic maps in monolinguals **(A)** and bilinguals **(B)**, with –200–0 ms pre-stimulus interval as baseline. (SP, the strongly constraining/high-cloze, plausible condition; SI, the strongly constraining/implausible condition; WP, the weakly constraining/low-cloze, plausible condition; WI, the weakly constraining/implausible condition). Negative is plotted down.

**FIGURE 3:**
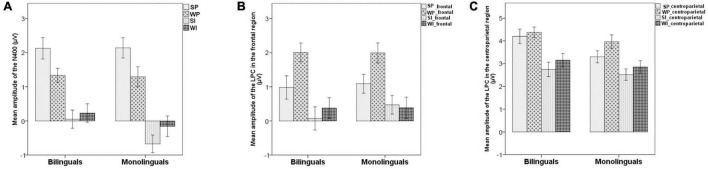
Mean amplitudes of the N400 (300–500 ms) in **(A)** and the LPC (500–700 ms) in for critical conditions over frontal **(B)** and centro-parietal **(C)** regions in monolinguals and bilinguals. (SP, the strongly constraining/high-cloze, plausible condition; SI, the strongly constraining/implausible condition; WP, the weakly constraining/low-cloze, plausible condition; WI, the weakly constraining/implausible condition). Error bars represent standard deviation.

Grand-averaged ERP waveforms and N400 results are shown in Figures 2, 3. The initial two-way ANOVA only revealed a main effect of Condition [*F*(3,168) = 31.960, *p* < 0.001, $\eta_{\text{p}}^{2}$ = 0.363]. Neither the main effect of Group [*F*(1,56) = 0.171, *p* = 0.680, $\eta_{\text{p}}^{2}$ = 0.003] nor the Condition × Group interaction [*F*(3,168) = 0.726, *p* = 0.532, $\eta_{\text{p}}^{2}$ = 0.013] was significant. Pairwise comparisons with Bonferroni correction indicated an N400 pattern in form of WI/SI > WP > SP (WI vs. SI: *p* = 1; WI vs. WP: *p* < 0.001; WI vs. SP: *p* < 0.001; SI vs. WP: *p* < 0.001; SI vs. SP: *p* < 0.001; WP vs. SP: *p* = 0.025; > means larger N400 amplitudes) across groups over the centro-parietal region.

#### The Late Positive Complex

Grand-averaged ERP waveforms and LPC results at representative sites to critical nouns are shown in Figures 2, 3. The initial three-way ANOVA revealed a main effect of Condition [*F*(3,168) = 13.881, *p* < 0.001, $\eta_{\text{p}}^{2}$ = 0.199], a main effect of ROI [*F*(1,56) = 153.201, *p* < 0.001, $\eta_{\text{p}}^{2}$ = 0.732], and a Condition × ROI interaction [*F*(3,168) = 3.631, *p* = 0.017, $\eta_{\text{p}}^{2}$ = 0.061]. No other significant main effect [Group: *F*(1,56) = 0.070, *p* = 0.793, $\eta_{\text{p}}^{2}$ = 0.001] or interactions with Condition [ROI × Group: *F*(1,56) = 2.142, *p* = 0.149, $\eta_{\text{p}}^{2}$ = 0.037; Condition × Group: *F*(3,168) = 0.252, *p* = 0.852, $\eta_{\text{p}}^{2}$ = 0.004; Condition × ROI × Group: *F*(3,168) = 1.449, *p* = 0.233, $\eta_{\text{p}}^{2}$ = 0.025] were significant. Follow up analyses within each ROI revealed a main effect of Condition across groups in both the frontal [*F*(3,171) = 13.809, *p* < 0.001, $\eta_{\text{p}}^{2}$ = 0.195] and centro-parietal [*F*(3,171) = 11.895, *p* < 0.001, $\eta_{\text{p}}^{2}$ = 0.173] ROIs. Pairwise comparisons with Bonferroni correction indicated a frontal positivity across groups in response to the WP condition compared to the SP, WI, and SI conditions (WP vs. SP: *p* = 0.007; WP vs. WI: *p* < 0.001; WP vs. SI: *p* < 0.001), while no significant differences between other three conditions were observed (SP vs. SI: *p* = 0.088; SP vs. WI: *p* = 0.287; WI vs. SI: *p* = 1). This effect extended into the centro-parietal ROI, with a larger positive-going effect in response to the WP condition compared to the WI (*p* = 0.001) and SI (*p* < 0.001) conditions. In addition, there was a larger positivity in response to the SP condition compared to the SI condition in the centro-parietal ROI (*p* = 0.002).

### Global Field Power Data

**FIGURE 4:**
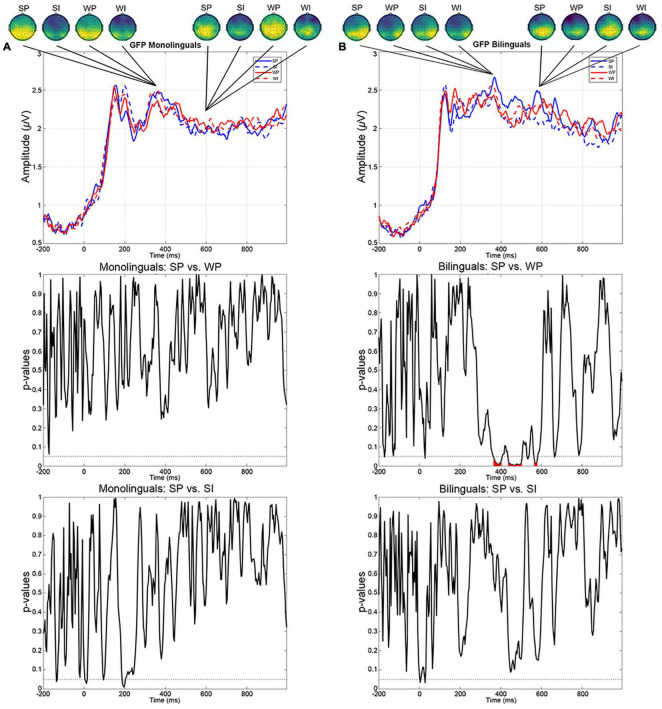
Global field power (GFP) data obtained from the grand-mean ERP for the four conditions (SP, WP, SI, and WI), as well as pairwise comparisons (SP vs. WP, SP vs. SI) in monolinguals **(A)** and bilinguals **(B)**. GFP differences between conditions within the N400 and the LPC time windows are highlighted. Scalp potential topography was added to depict the ERP polarity for the GFP peaks.

The GFP curve of the grand mean electric potential across participants for each condition and for each group, as well as the comparison between SP and WP, and the comparison between SP and SI as representative of plausible vs. implausible contrasts are shown in Figure 4. Results of GFP analysis showed significant differences between the SP and WP conditions in the bilingual group at 375–410, 440–510, and 565-585 ms, indicating a stronger GFP in response to the SP condition compared to the WP condition. In contrast, this difference failed to reach statistical significance in the monolingual group. No other significant differences between conditions were observed in both mono- and bilingual groups.

## Discussion

The aim of the current study was to investigate how heritage Chinese speakers process lexico-semantic aspects of written language and how their brain patterns compare to those of monolingual Chinese speakers. Our hypotheses were based on a previous ERP study of us examining N400 and LPC waves (Li et al., 2021). With respect to both ERP components, we did not find any significant group differences. Below we discuss the results by the N400 and the LPC in turn.

Across the groups, a graded N400 response with a pattern in form of WI = SI > WP > SP was observed at the critical noun, modulated by semantic congruencies between classifiers and nouns. Consistent with findings from other studies (e.g., Federmeier et al., 2007), and as indicated in our previous study (Li et al., 2021), we did not find any significant N400 differences between the SI and WI conditions in monolinguals, suggesting that the N400 is not sensitive to contextual constraints when target nouns are implausible. This result is consistent with the findings of Federmeier et al. (2007) with sentential contexts. It would be interpreted as the fact that the N400 reflects context-based facilitation rather than processing costs when a predicted target stimulus (e.g., in a strongly constraining context) is not encountered (see Van Petten and Luka, 2012, for a review). In the present study, our results showed that the same effect can be observed in bilinguals. Some researchers (Hahne and Friederici, 2001; Moreno and Kutas, 2005; Moreno et al., 2010) interpreted the similarity on processing semantic anomalies as evidence for that bilinguals engaged approximately equivalent processing resources to the task of appreciating the semantic relation between the target word and its prior context. In contrast, McLaughlin et al. (2004) showed a strong, native-like N400 peak for semantic anomalies after only a 9-month instructional period in an L2, indicating that the N400 semantic anomaly effect is involved at the earliest stage of L2 learning. Thus, semantic anomalies may not be suitable indicator of language expose and language proficiency.

More importantly, the N400 amplitude did not differ between groups significantly in response to the WP condition, indicating bilinguals’ sensitivity to subtle manipulations of semantic fit in our study. According to current evidence (see section “Introduction”), increased N400 amplitudes in bilinguals reflect less efficient or failed retrieval of conceptual knowledge with the eliciting word form from long-term semantic memory and the effort involved in integrating the meaning of an eliciting target noun with its preceding context. And, previous research indicates that less efficient lexical access was often observed in late bilinguals who learn an L2 after puberty (Ardal et al., 1990; Weber-Fox and Neville, 1996; Moreno and Kutas, 2005). Our results are consistent with other studies with early bilinguals or highly proficient bilinguals as target groups (Weber-Fox and Neville, 1996; Braunstein et al., 2012; Kasparian and Steinhauer, 2016) showing that the heritage speakers in our study were highly proficient Chinese speakers.

In terms of the LPC, we also did not observe any group effects. Across the mono- and bilingual groups, we found a frontally-distributed positive shift in response to the WP condition, with some extension into the centro-parietal region. The scalp distribution of the LPC effect differed from the posterior P600 effect reported by previous studies for strong semantic violations (Kolk et al., 2003; Kim and Osterhout, 2005; Van Herten et al., 2006; Kuperberg, 2007; Bornkessel-Schlesewsky and Schlesewsky, 2008; Van De Meerendonk et al., 2010). It resembled more closely that of frontocentral LPC related to increased conflicts triggered by prediction violations (Federmeier et al., 2007; Kutas et al., 2011; Thornhill and Van Petten, 2012; Van Petten and Luka, 2012), or post-lexical semantic integration difficulty by encountering unexpected but plausible target words (Molinaro et al., 2012; Brothers et al., 2015). Available functional neuroimaging evidence (Wagner et al., 2001; Badre and Wagner, 2002) also support the relevance of the frontal cortex in controlled retrieval/selection of semantic representations based on context. According to Lau et al. (2008), observed effects in those areas in imaging studies can be associated with the frontal post-N400 positivity, reflecting more effortful selection or inhibition relative to more posteriorly-dominant effects. Our finding adds to the already substantial body of evidence showing that prediction is a mechanism that contributes to language processing in various contexts and under various circumstance, including bilingual processing (Kaan, 2014; Kuperberg and Jaeger, 2016). It is worth mentioning that, in our study, bilinguals showed less certainty in their behavioral responses to the SP and WP conditions compared to monolinguals, whereas LPC amplitudes did not show group differences in response to both conditions. The relationship between LPC effects in online processing vs. behavioral responses therefore remains an open question.

At the same time, a larger centro-parietal LPC observed in response to the SP condition compared to the SI condition across groups highlights the observation that the LPC, at least in classifier-noun phrase processing, reflects some form of combinatorial processing rather than a single isolated process. According to previous research, acceptability judgment tasks might involve more attention or cognitive resources devoted to semantic deviance in order to provide task-relevant information allowing for a negative decision, typically leading to an enhancement of the late “wrap-up” positivity (Kolk et al., 2003; Schacht et al., 2014). The “wrap-up” positivity is considered to reflect the retrospective, evaluative processing by the time the last word of a sentence or a clause is perceived. It has a centro-parietal distribution, and typically starts in the N400 time window (300–500 ms), and is long-lasting (see Stowe et al., 2018, for a review). Thus, it is reasonable to hypothesize that selecting which classifier modifies a noun in a less reliable information source manner would engender a second-pass reanalysis, perhaps, at least partially, triggered by some type of “phrase wrap-up” effect in attempt to assign a full interpretation to the expression. As such, it is possible that the larger centro-parietal LPC effects observed in response to the WP condition compared to the WI and SI conditions reflect fine grained wrap-up demands provided by conditions, rather than an extended frontal positivity into centro-parietal areas due to volume conduction. Further studies are needed to better differentiate these effects.

In addition, we did not find any LPC differences between groups in response to the implausible WI and SI conditions. A possible reason for the absence of group differences is that the task in our study was relatively simple. The participants read simple classifier-noun phrases, with the classifier serving as the prime and the noun as the target. The memory load was low, and so was the reanalysis demands during processing implausible combinations. Indeed, past research has shown that differences between monolingual speakers and heritage speakers in the late posterior LPC are often observed in tasks requiring in-depth and continued integrative efforts, such as comprehension of sentence-level information, and outright semantic anomalies when the prior context is rich (e.g., Kasparian and Steinhauer, 2016; Zheng and Lemhöfer, 2019).

The ERP and GFP data did not yield consistent results in the N400-LPC analysis with regard to the plausible vs. implausible contrast. Because GFP calculation reflects differences in strength of the electric field rather than differences in the configuration of ERP amplitudes, it is not unexpected to observe inconsistent results between ERP data and GPF data. Interestingly, the GFP data showed differences for the composition effect in response to the SP and WP conditions in the bilingual group, while no such effect was observed in the monolingual group. This effect began within the N400 range and extended into the late 500–600 ms time window. Because differences in GFP are attributable to differences in the amount of synchronized neuronal activation, this effect can be interpreted in terms of stronger engagement of neuronal resources in response to the SP condition compared to the WP condition during semantic processing in bilinguals. One possible explanation may have to do with the fact that, compared to monolinguals, even the SP condition that involved the largest amount of typical and preferred combinations required outright attention and a more explicit metalinguistic analysis in bilinguals. Another possible explanation is that bilingual speakers have generally a more developed monitoring system than monolingual speakers (Costa et al., 2008; Schmid and Köpke, 2009; Kroll and Bialystok, 2013; Grant et al., 2014; Duncan et al., 2016). According to this assumption, bidirectional cross-linguistic adaptation occurs at any time on multiple levels during language processing in the bilingual brain. This makes bilingual language processing less efficient as a result of added task demands. Because cognitive resources are limited, bilinguals may develop more efficient strategies of attention control in both L1 and L2, in order to achieve a certain degree of processing efficiency. This eventually resulted in bilingual inhibitory control advantage and/or a bilingual advantage for attentional monitoring, especially when a word is in the focus of attention and when strong competitors are activated, as in the SP condition (see Van Petten and Luka, 2012, for a review). The effects of bilingualism in monitoring and resolving conflicting information may be the key point of L1 attrition, as emphasized by Kasparian and Steinhauer (2016), or differences in strategies to process the semantic information online. Specific studies and methods addressing this point are needed.

### Caveats and Limitations in Relation to the Study

Although group differences in relation to the N400/LPC pattern were non-significant, it is not possible to exclude the possibility that both the N400 and the LPC may still be relevant with regard to individual differences in bilinguals, and that group differences in relation to the N400/LPC pattern would have been discernable (i.e., met the standard threshold of statistical significance) were the sample size larger. The possible presence of separate strategies to process semantic information online between groups is, at least indirectly, supported by the GFP data. It should be noted that our bilingual data indicate a robust and replicable N400/LPC congruency pattern during processing Chinese classifier-noun phrases in bilinguals. Nonetheless, it would be advantageous for future investigations to recruit more potential eligible participants and involve multiple sessions and levels of psychophysiological and language testing.

Previous studies suggested that bilinguals simultaneously access words from their other language as well, when they perform a task in one of their languages (see van Heuven and Dijkstra, 2010, for a review). The parallel activation of lexicons in different languages is thought to give rise to between-language competition that imposes demands on the bilingual to control the language not in use to achieve fluency in the target language, resulting in weaker access to a given target word. Cross-linguistic interference occurs most likely between two typologically similar languages (Połczyńska and Bookheimer, 2020). Although Chinese (Sino-Tibetan) and Malay (Austronesian) are two typologically distant languages, both languages, however, are classifier languages which invoke the noun classifiers (Aikhenvald, 2006). Consequently, the learners of classifier languages are presented with an intriguing interference point as part of their language learning experience. Since the present study has not investigated cross-language interference, language-selective access and degree of top-down control of bilingual lexical processing *per se*, further studies with more fine-grained manipulations are needed.

## Conclusion

The present ERP study examined whether a previously observed neural signature in monolingual speakers in relation to Chinese classifier-noun phrase processing was present in Chinese-Malay bilingual speakers. The findings confirmed the importance of two ERPs, the N400 and a post-N400 LPC, in indexing semantic congruencies within classifier-noun phrases in both mono- and bilingual groups. Our results showed that no group differences were seen in semantic congruency effects, neither in the response pattern within the N400, which primarily indexes lexical access and first-pass semantic integration, nor in the post-N400 LPC component, which is believed to primarily index second-pass, attention-driven, integration processes after prediction violations. Our results suggest that, in terms of the ERP data, Chinese-Malay heritage speakers predict and integrate upcoming semantic information in Chinese classifier-noun phrase to the same extent as monolingual Chinese speakers. Furthermore, our GFP data showed that bilinguals differ from their monolingual peers in terms of global field power intensity of the brain by processing plausible classifier-noun pairs with different congruency effects. Further studies are needed to clarify this finding. The present study is only a first step in comparing online lexico-semantic processing in Chinese heritage speakers with Chinese monolingual speakers. We stated with reasonable confidence that Chinese-Malay bilinguals are clearly the population of interest for the research on neuroplasticity in the bilingual brain.

## Data Availability Statement

The original contributions presented in the study are included in the article/Supplementary Material, further inquiries can be directed to the corresponding author.

## Ethics Statement

The studies involving human participants were reviewed and approved by the Ethics Committee of School of Foreign Languages, Shanghai Jiao Tong University. The patients/participants provided their written informed consent to participate in this study.

## Author Contributions

FL and XH collaborated in specifying the experimental design and participated in data analyses. FL, ZH, SW, and CZ prepared experimental materials and collected data. FL, XH, and ZH contributed to the writing of the article. All authors contributed to the article and approved the submitted version.

## Conflict of Interest

The authors declare that the research was conducted in the absence of any commercial or financial relationships that could be construed as a potential conflict of interest.

## Publisher’s Note

All claims expressed in this article are solely those of the authors and do not necessarily represent those of their affiliated organizations, or those of the publisher, the editors and the reviewers. Any product that may be evaluated in this article, or claim that may be made by its manufacturer, is not guaranteed or endorsed by the publisher.

## Footnotes

1
https://osf.io/eptqz/
