# Supplementary material for: Investigating Heritage Language Processing: Meaning Composition in Chinese Classifier-Noun Phrasal Contexts
Source: Front Psychol. 2021 Dec 17;12:782016. doi: 10.3389/fpsyg.2021.782016 (PMC8718634; doi:10.3389/fpsyg.2021.782016)
Supplement: Supplementary file 4 [file Data_Sheet_4.DOCX]

Supplement 1. The HSK Level 5 test

The HSK Level 5 is intended for students who have mastered over 2500 commonly used words, and is the counterpart of the C1 Level of the Common European Framework of Reference (Teng, 2017). For the HSK Level 5, four results are provided including listening (max. score: 100), reading (max. score: 100), writing (max. score: 100), and total (max. score: 300). Test takers must score at least 180 points total to be considered passing.

Teng, Y. (2017). Hanyu Shuiping Kaoshi (HSK): Past, Present, and Future. In D. Zhang & C.-H. Lin (Eds.), Chinese as a Second Language Assessment (pp. 3–19). <https://doi.org/10.1007/978-981-10-4089-4>

Supplement 2. Materials and design

To determine the strength of constraint for classifiers and the cloze probability for nouns, a norming procedure was conducted with 57 Mandarin Chinese speakers (46 aged 18-40 years, 11 aged over 40 years old; 30 females) who did not participate in the EEG experiment. For rating strength of constraint, participants were asked to perform a subjective rating on a 5-point Likert-type scale from 1 (could not think only one noun or could not think of any noun) to 5 (more than four nouns). The strength of the constraint was calculated through average of the rating (mean = 3.31, SD = 0.68). For norming the cloze probability, participants were asked to provide all of the nouns that could be preceded by each classifier, if possible, as well as the possibility of classifier-noun pairs corresponding to the order in which the nouns are provided in the list. To calculate the cloze probability, the following formula was used: the degree of cloze probability equals the number of target tokens divided by the sum of tokens for a specific classifier-noun pair.

Based on the norming results, strongly or weakly constraining classifiers were defined by whether the constraining strength was less than or more than 3.2, as determined by a median cut-off. In addition, the high-cloze, plausible nouns were the most frequently chosen words in the questionnaire, while the low-cloze, plausible nouns were the nouns that were rarely chosen by the questionnaire participants. For the implausible conditions, anomalous nouns that did not appear in the blank question were used. To calculate the cloze scores for implausible items, the same formula in the plausible condition was applied. To ensure the unacceptability of the implausible pairs, fifty-eight independent raters (50 aged 18-40 years, 8 aged over 40 years; 35 females) were asked to score all implausible classifier-noun pairs offline for acceptability on a scale of 1 (totally unacceptable) to 7 (perfectly acceptable). The results showed that the implausible classifier-noun pairs were totally unacceptable (mean = 1.05, SD = 0.07). We note that because we applied a more stringent criterion for selecting classifiers (matched for classifier frequency, noun frequency, the number of classifier strokes, and the number of noun strokes) compared to previous studies with similar designs (e.g., Chou, Huang, Lee, & Lee, 2014), the two variables (i.e., “constraint” and “cloze probability”) were not orthogonal to each other in the present study.

In addition to 144 classifier-noun pairs, another 432 fillers in form of word triplets were added. For these fillers, half of the pairs were semantically plausible and the other half were semantically implausible. Fillers were added to prevent participants from focusing on the classifier noun phrases. To avoid repetition effect of the classifiers, plausible and implausible conditions of each classifier-noun pair were counterbalanced in terms of their sequences in stimuli presentation, i.e., for the same classifiers, half of the plausible pairs preceded their corresponding implausible pairs, the other half appeared in a reversed sequence.
